# Assessments of Total and Viable *Escherichia coli* O157:H7 on Field and Laboratory Grown Lettuce

**DOI:** 10.1371/journal.pone.0070643

**Published:** 2013-07-30

**Authors:** Anne-Laure Moyne, Linda J. Harris, Maria L. Marco

**Affiliations:** 1 Department of Food Science and Technology, University of California Davis, Davis, California, United States of America; 2 Western Center for Food Safety, University of California Davis, Davis, California, United States of America; U. S. Salinity Lab, United States of America

## Abstract

Leafy green produce has been associated with numerous outbreaks of foodborne illness caused by strains of *Escherichia coli* O157:H7. While the amounts of culturable *E. coli* O157:H7 rapidly decline after introduction onto lettuce in the field, it remains to be determined whether the reduction in cell numbers is due to losses in cell viability, cell injury and a subsequent inability to be detected by standard laboratory culturing methods, or a lack of adherence and hence rapid removal of the organism from the plants during application. To assess which of these options is most relevant for *E. coli* O157:H7 on leafy green produce, we developed and applied a propidium monoazide (PMA) real-time PCR assay to quantify viable (with PMA) and total (without PMA) *E. coli* O157:H7 cells on growth chamber and field-grown lettuce. *E. coli* O157:H7, suspended in 0.1% peptone, was inoculated onto 4-week-old lettuce plants at a level of approximately 10^6^ CFU/plant. In the growth chamber at low relative humidity (30%), culturable amounts of the nontoxigenic *E. coli* O157:H7 strain ATCC 700728 and the virulent strain EC4045 declined 100 to 1000-fold in 24 h. Fewer *E. coli* O157:H7 cells survived when applied onto plants in droplets with a pipette compared with a fine spray inoculation. Total cells for both strains were equivalent to inoculum levels for 7 days after application, and viable cell quantities determined by PMA real-time PCR were approximately 10^4^ greater than found by colony enumeration. Within 2 h after application onto plants in the field, the number of culturable *E. coli* ATCC 700728 was reduced by up to 1000-fold, whereas PCR-based assessments showed that total cell amounts were equivalent to inoculum levels. These findings show that shortly after inoculation onto plants, the majority of *E. coli* O157:H7 cells either die or are no longer culturable.

## Introduction

Fresh and minimally processed fruit and vegetables contribute significantly to the burden of foodborne illness in the U.S. [1]. While introduction of pathogens can occur at any step from production through consumption, preharvest contamination of leafy greens has been identified in a number of recent outbreaks [2], [3]. Recurrent outbreaks and product recalls associated with leafy greens and the isolation of *Salmonella* or *Escherichia coli* O157:H7 from implicated product suggest that specific plant-bacteria interactions or environmental conditions might favor the introduction and persistence of these pathogens in the production environment.

To evaluate the risks of preharvest contamination of leafy greens, the ability of *E. coli* O157:H7 to colonize or survive on plants has been assessed under both laboratory and field conditions. On plants in growth chambers, *E. coli* O157:H7 was found to grow and survive under specific conditions on lettuce seedlings and mature plants [4]–[8]. Growth of *E. coli* O157:H7 was generally associated with the phyllosphere, or the aerial portions of plants [9], [10]. Endophytic growth has also been demonstrated, but mainly for plants in hydroponic solution [10]. Young lettuce leaves were found to sustain higher populations of the pathogen than older leaves [11].

Although controlled conditions in the laboratory provide an opportunity to study virulent strains of human pathogens, such investigations do not fully mimic the (a)biotic conditions plants are exposed to in the field. Shortly after application of (non-toxigenic) *E. coli* O157:H7 onto lettuce in production regions, culturable populations of the pathogen rapidly declined [12]–[14], regardless of the season [14] or location (e.g. Salinas Valley, California, USA [14]; Tifton, Georgia, USA [12]; or Summerland, B.C., and Kentville, Nova Scotia, Canada [13]). However, *E. coli* O157:H7 can apparently survive, at least in low quantities, on field-grown plants, and viable cells were found on a fraction of lettuce plants for several weeks after inoculation [12]–[15].

Studies of *E. coli* O157:H7 survival on plants in the field have generally relied on colony-based assessments coupled with enrichment methods to detect the presence/absence of the pathogen. Such approaches do not permit measurements of total (viable and non-viable) cell amounts or the detection of individual cells that are viable but injured and no longer recovered on standard laboratory growth medium. These sub-lethally injured cells may still be infectious or capable of resuming growth under favorable conditions. The latter possibility is relevant to leaf surface habitats, which are characterized as stressful environments for microorganisms and are subject to fluctuating temperatures and moisture levels as well as other environmental insults [16]. Therefore, culture-independent methods, such as fluorescence-based cell detection or genetic approaches, might be complementary for measurements of pathogen presence and survival on plants. In particular, combining membrane-impermeable, DNA-intercalating compounds, such as propidium monoazide (PMA), with quantitative real-time PCR might offer novel opportunities to sensitively and selectively detect pathogens among a background of other microorganisms in environmental samples [17]. PMA efficiently enters cells with compromised but not intact membranes, and, upon photoactivation, PMA is irreversibly cross-linked to DNA and prevents PCR amplification of target sequences.

In this study, we developed a PMA real-time PCR assay to quantify both viable and total *E. coli* O157:H7 recovered from lettuce plants in the laboratory and field; these results were compared with quantities of this organism determined by culture methods. We also used this approach to design plant inoculation and incubation conditions in the laboratory that more closely mimic field environments and could be used to study the plant-associated behaviors of virulent outbreak-associated strains of the pathogen.

## Materials and Methods

### Bacterial Strains and Culture Conditions

*E. coli* O157:H7 strain EC4045 was kindly provided by Thomas A. Cebula (John Hopkins University, USA). This strain was isolated from spinach during a 2006 *E. coli* O157:H7 spinach-associated outbreak [18]. A spontaneous rifampicin-resistant mutant of EC4045 was isolated by selecting colonies resistant to 120 µg/ml rifampicin (Gold Biotechnology, St. Louis, MO). Rifampicin-resistant *E. coli* O157:H7 ATCC 700728 lacks *stx1* and *stx2* genes and was used in previous field trials [14]. The cells were routinely cultured on tryptic soy broth (TSB) or tryptic soy agar (TSA) from BD (Franklin Lakes, USA) at 37°C. For selective growth of *E. coli* O157:H7 strains ATCC 700728 and EC4045, rifampicin was added to the medium at 50 µg/ml.

To prepare the *E. coli* O157:H7 cells for inoculation onto plants, 20 µl of an overnight bacterial liquid culture was spread plated with an automated spiral plater (Autoplate 4000, Spiral Biotech Inc., Boston, MA) onto TSA and incubated at 37°C for 12 h to produce a bacterial lawn. Cells were dislodged with 5 ml of 0.1% peptone, and then the suspension was diluted in 0.1% peptone to the desired concentration.

### Inoculation of *E. coli* O157:H7 onto Lettuce Grown in the Laboratory

Seeds of Romaine lettuce (*Lactuca sativa*) cv. Parris Island were planted in Sunshine mix potting soil (Sun Gro Horticulture Distribution, Bellevue, WA) and grown in an environmental chamber (PGR15, Conviron, Pembina, ND) with a light intensity of 230 µmol m^−2^ s^−2^. The chamber was maintained at a constant relative humidity (60% RH) and daily temperatures of 22°C (for 12 h, with light) and 18°C (for 12 h, without light). Starting 2 weeks after emergence, the plants were fertilized weekly with Hoagland nutrient water.

Four weeks after plant emergence, 10 2-µl drops of *E. coli* O157:H7 ATCC 700728 or EC4045 were inoculated onto the leaves. The bacterial suspension was dispensed with an Eppendorf repeater pipette (Eppendorf, Hauppauge, NY) onto the interveinal spaces of two leaves per plant to yield approximately 10^6^ CFU/leaf. *E. coli* O157:H7 ATCC 700728 was also inoculated onto lettuce plants using a spray bottle as described previously for field studies [14]. Spray bottles were calibrated to deliver a dose adjusted to 10^6^ CFU of *E. coli* O157:H7 ATCC700728 per spray, and a single spray was applied to each plant from a distance of 20 cm and with a dispersal area of approximately 0.3 m^2^. Immediately after inoculation, plants were transferred to a growth chamber (Percival, Geneva Scientific LLC, Fontana, WI) maintained with a 12-h photoperiod (light intensity 230 µmol m^−2^ s^−2^), constant relative humidity (30% RH), and temperatures of 18°C (12 h) and 23°C (12 h). Within 24 h after application, free water was no longer visible on the surface of the plants. Cross-contamination of strains EC4045 and ATCC 700728 was prevented by inoculating one strain per plant and keeping plants spatially separated. Five leaves (drop inoculation) or five plants (spray inoculation) were collected for measurement of *E. coli* O157:H7 amounts at the time of inoculation and then 1, 2 and 7 days after application of the pathogen onto the plants.

### Field Trials

Field trials were conducted in the Salinas Valley during spring 2010 (5/4/10 to 7/5/10) and summer 2010 (7/28/10 to 10/4/10). Permits and approvals for use of United States-owned land were granted by the United States Department of Agriculture. All beds were direct seeded according to standard commercial practice with two rows of Romaine lettuce (*Lactuca sativa*) cv. Green Towers per bed. The field trials were conducted exactly as described in Moyne et al. [14] on 4-week old lettuce plants after thinning. Inoculation was carried out in the morning (when no dew was visible) by delivering one spray (10^7^ CFU) per 4-week-old lettuce plant just after thinning. For *E. coli* O157:H7 enumeration on lettuce, entire lettuce heads were removed by cutting at the base with a sterile scalpel approximately 3 cm above the ground; each head was bagged individually at the time of collection. Twelve lettuce heads were sampled immediately after inoculation, and 24 lettuce plants were then randomly sampled among the inoculated beds at 2 h and 2 and 7 days. Twenty-four non-inoculated lettuce heads (controls) were collected concurrently at the time of inoculation. The lettuce was brought to the laboratory from the field in a cooler on ice and analyzed within 24 h.

### Enumeration of Culturable *E. coli* O157:H7 Populations on Lettuce

One leaf (laboratory drop-inoculated sample) or the entire lettuce head (laboratory spray-inoculated and field-inoculated sample) was placed into a sterile Whirl-Pak bag (Nasco, Modesto, CA) with 50 ml of 0.1% peptone water. The lettuce was homogenized in a Stomacher 400 laboratory blender (Seward, Westbury, NY) at medium speed for 2 min. A total of 500 µl of the resulting cell suspension was used for PMA real-time PCR and 1 ml was serial diluted for CFU enumerations on TSA supplemented with 50 µg/ml rifampicin. To improve the limit of detection of culturable cells, the remaining cell suspension was filtered through disposable analytical filter units (0.45 µm; Nalgene, Rochester, NY). Filter membranes were then laid onto CHROMagar O157 (CHROMagar, Paris, France) [19] and incubated at 37°C overnight. The detection limit by this approach was 1 CFU per leaf or plant in the laboratory experiments and 10 CFU per plant in the field experiments. In addition to plating for CFU enumeration, the lettuce heads were mixed at a ratio of 1∶2 (wt lettuce:vol culture medium) with TSB supplemented with rifampicin at 50 µg/ml and incubated at 42°C for 18 h. When CFU estimates were low, the enrichment broth culture was spiral plated onto CHROMagar O157 supplemented rifampicin at 50 mg/ml and incubated at 37°C overnight. The enrichment was considered positive if mauve colonies typical of *E. coli* O157:H7 on this medium were observed.

### PMA Assay, DNA Extractions, and Real-time PCR Quantification

PMA (Biotum, Hayward, CA) was dissolved in water to obtain a 20 mM stock solution and stored at −20°C in the dark. For comparisons of different PMA concentrations, 40, 50, or 100 µM PMA was added to 500 µl cells of *E. coli* O157:H7 in clear transparent 2-ml microtubes and incubated, with shaking at 400 rpm, at room temperature in the dark for 15, 30, or 60 min. After incubation with PMA, samples were exposed to a 500W halogen light at a distance of 20 cm for 3 min with occasional mixing. To avoid overheating, the cultures were kept on ice during light exposure.

Assessment of PMA for distinguishing live and dead cells were performed by incubating one half of a 12-h *E. coli* O157:H7 culture in 0.1% peptone (live) and suspending the other cell fraction in 70% isopropanol for 10 min (dead). Both live and dead fractions were then washed twice in 0.1% peptone and suspended in 0.1% peptone for exposure to PMA.

DNA was extracted from cells recovered from laboratory cultures and lettuce with or without prior exposure to PMA. First, 500 µl of the cell suspension was concentrated by centrifugation at 10,000×*g* for 2 min. The resulting cell pellet was then washed once with 0.1% peptone, suspended in 100 µl of Prepman solution (Applied Biosystems, Foster City, CA), and boiled for 5 min. Debris was removed by centrifugation at 10,000×*g* for 2 min, and 1 µl of the supernatant was used for real-time PCR. High quality *E. coli* O157:H7 genomic DNA for use in the real-time PCR assay development was obtained using the Wizard Genomic Purification kit (Promega, Madison, WI) as described by the manufacturer.

Real-time PCR was performed on an ABI 7500 Fast Real-Time PCR system (Applied Biosystems) using 0.25 µM of forward and reverse primers, 1X Ssofast Evagreen Supermix (Bio-Rad, Hercules, CA) and 1 µl of prepared DNA template. Cycling conditions included an initial activation step at 95°C for 10 s, followed by 40 cycles of denaturation at 95°C for 5 s, and annealing/extension temperatures between 60 to 68°C for 30 s [20], [21] for the primers shown in Table S1, supporting information. After 40 PCR cycles, melting curves were generated by increasing the temperature from 60 to 95°C at 0.2°C/10 s and recording the fluorescence. Threshold cycle (Ct) values were automatically generated by the 7500 Fast Real-Time PCR software.

To quantify the number of *E. coli* O157:H7 cells in the samples, a standard curve was included for each plate with three replicates for each DNA concentration. Standard curves were generated by amplifying a 5-log DNA dilution series from a known concentration of *E. coli* O157:H7 cells.

### Statistical Analysis

Microbial data (CFU per plant or leaf) were log transformed before being statistically analyzed with JMP software (SAS Institute Inc., Cary, NC). Analysis of variance (ANOVA) was performed to compare the survival of *E. coli* O157:H7 strains at each time point after inoculation. Differences between means were considered significant at *P*<0.05.

## Results

### Development of PMA Real-time PCR Assay for Quantification of Live and Dead *E. coli* O157:H7 Cells on Lettuce

The use of PMA to prevent amplification of target DNA from dead cells in real-time PCR assays has been applied successfully for a variety of organisms and environments [22]–[27]. However, PMA real-time PCR assays should be optimized for each strain and condition of interest. Therefore, we investigated several parameters for quantification of low amounts of viable and total *E. coli* O157:H7 cells on lettuce.

#### Primer selection for E. coli ATCC 700728

The *stx1* or *stx2* genes coding for Shiga-toxin 1 and 2 are typically used as genetic targets for detection or quantification of *E. coli* O157:H7. Because these genes are absent from the nontoxigenic strain ATCC 700728 and a genome sequence for this strain was not available, real-time PCR primers designed for other genes in *E. coli* O157:H7 were compared including enterocyte effacement loci genes *csgA* (curli fimbriae), *eae* (intimin), *espA* (extracellular protein), *fliC* (flagellar antigen), *ler* (positive regulator of LEE), and *lpfA* (long polar fimbriae) [20] (Tables S1 and S2, supporting information).

Our goal was to develop a method capable of detecting small amounts of *E. coli* O157:H7 ATCC 700728 cells among high numbers of endogenous bacteria present on lettuce leaves (between 10^4^ to 10^5^ cells/g [14]). Therefore, primers were selected based on minimum Ct (a measure of sensitivity) for amplifying 1 ng of *E. coli* O157:H7 ATCC 700728 genomic DNA (the equivalent of 10^5^ genomes) and maximum Ct (a measure of selectivity) for amplifying 1 ng of DNA isolated from the closely-related bacterial strain *E. coli* K12. These comparisons were performed because non-pathogenic *E. coli* were detected on some of the lettuce heads sampled in previous field studies [14], albeit in much lower amounts than evaluated in this assay.

*E. coli* K12 DNA was amplified in low quantities with all primer sets when the annealing temperature was less than 65°C (Table S2, supporting information). Although increasing the annealing temperature reduced the non-specific amplification of *E. coli* K12 DNA, the sensitivity of *E. coli* ATCC 700728 DNA detection also decreased (indicated by higher Ct values) and lowered the dynamic range of the assay (Table S2, supporting information). Based on these comparisons, we selected primers targeting the *lpfA* gene and an annealing temperature of 60°C because this combination resulted in the earliest detectable amplification (lowest Ct) of target *E. coli* O157:H7 ATCC 700728 genomic DNA and yielded the lowest levels of non-specific amplification (highest Ct) of *E. coli* K12 genomic DNA. The efficiency of the PCR with lpfA primers was between 92 to 100% and the correlation coefficient was between 0.98 and 0.99.

#### Optimization of PMA concentration and incubation time with cell cultures

**Figure 1:**
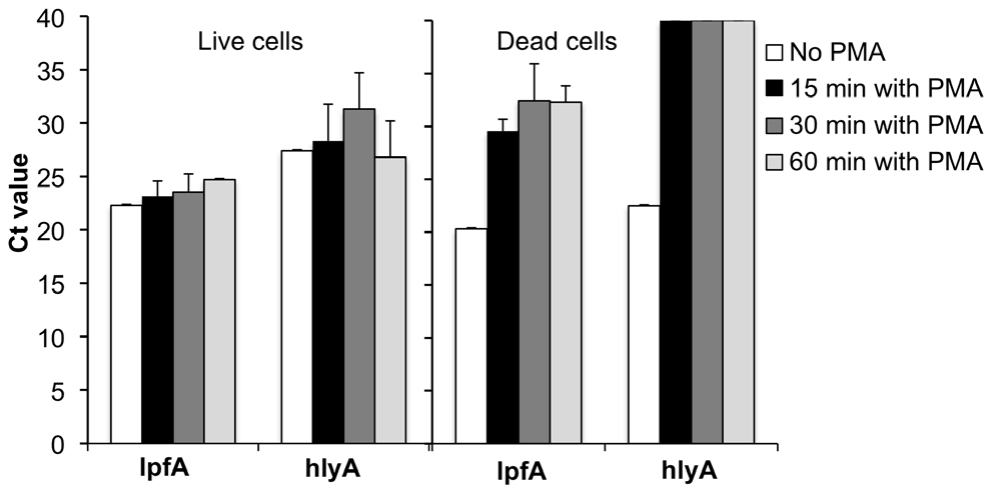
Effect of incubation time and PCR primer on detection of live and dead cells by real-time PCR. Live or isopropanol-inactivated dead cells of *E. coli* O157:H7 ATCC 700728 (5×10^7^ CFU) were incubated in the dark with or without 40 µM PMA for 15, 30, or 60. Real-time PCR was performed using the lpfA or hlyA primer sets [20], [21]. Each bar represents the mean ± stdev for three independent replicates.

To accurately quantify live and dead cells in a mixture, PMA should completely inhibit amplification of DNA from dead cells without affecting the enumeration of live cells. Real-time PCR amplification of nucleic acids from dead cells of *E. coli* O157:H7 ATCC 700728 was significantly reduced, but not completely inhibited, after treatment with PMA at concentrations of 50 and 100 µM, as indicated by an increase in the Ct values (Fig. S1, supporting information). These concentrations of PMA were high enough to influence the accurate detection of viable cells in the real-time PCR assay by increasing the Ct values for the viable cells. In contrast, PMA at a concentration of 40 µM did not interfere with DNA amplification from viable cells in the real-time PCR assay (Fig. 1). Amplification of DNA from dead cells was also significantly inhibited by over 9 Ct values at that concentration of PMA (Fig. 1). Increasing the exposure to PMA from 15 to 60 min yielded only modest effects (Fig. 1), and therefore, we used an incubation time of 30 min for subsequent studies.

To fully investigate the factors that resulted in albeit low, but still measurable, amounts of DNA amplification from dead cells after incubating in PMA, we examined the effects of long versus short PCR products on distinguishing cell viability. In contrast to the 165 bp PCR product resulting from the lfpA primers, PCR amplification was completely inhibited from ATCC 700728 cells using hlyA primers (528 bp product size) (Fig. 1). However, DNA amplification using the hlyA primers was 10-fold less sensitive for detecting low quantities of *E. coli* O157:H7 cells (Fig. S2A and S2B, supporting information). The limit of detection for both *E. coli* O157:H7 ATCC 700728 and EC4045 with the lpfA primers was 100 CFU/ml or 5000 CFU per plant or leaf (between 100 to 1000 CFU/g depending on the plant weight) (Fig. S2B, supporting information). Therefore, the lpfA primers were used in further studies for quantifying *E. coli* O157:H7 ATCC 700728 and EC4045 recovered from plants.

#### Interference of dead cells during viable cell quantification

**Figure 2:**
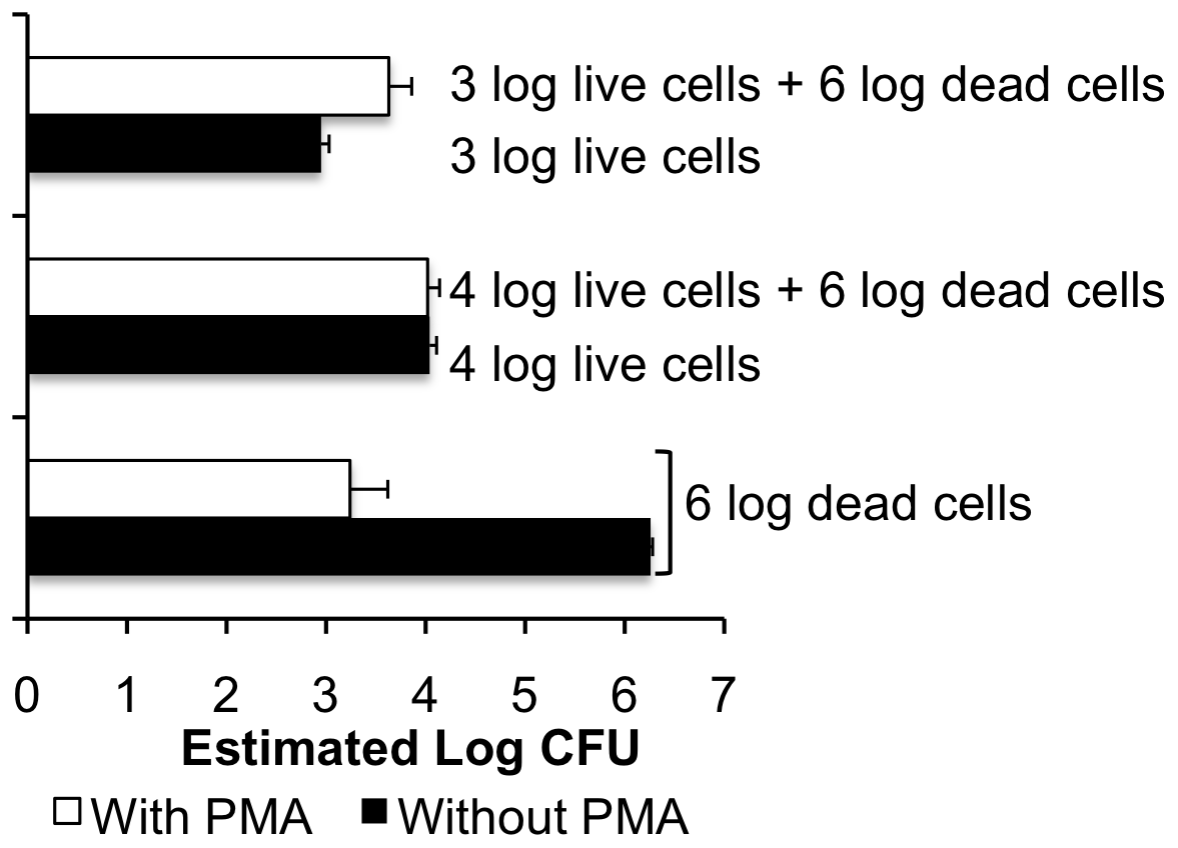
Interference of dead cells in the enumeration of live *E. coli* O157:H7 by PMA real-time PCR. Cell amounts were quantified by real-time PCR (total; black bars) or PMA real-time PCR (viable; open bars) in mixtures of 10^3^ or 10^4^ live cells mixed with 10^6^ isopropanol-inactivated dead cells. Samples were treated with 40 µM PMA for 30 min. Estimates of *E coli* O157:H7 cell amounts were based on comparisons to standard curves constructed using *E. coli* O157:H7 ATCC 700728 genomic DNA. Each bar represents the mean ± stdev for three independent replicates.

Previous studies have shown that PMA combined with real-time PCR is most accurate for viable cell enumeration when viable cells greatly outnumber dead cells [28]–[30]. In our field studies, *E. coli* O157:H7 ATCC 700728 populations recovered on laboratory culture media declined drastically shortly after inoculation of 10^6^ cells per lettuce head [14], establishing the potential for high numbers of dead cells that may interfere with quantification of the viable cells remaining on the plants. Indeed, suspensions of 10^6^ dead *E. coli* 0157:H7 ATCC 700728 cells were partially detected by PMA real-time PCR (estimated log 3 CFU; Fig. 2). In a mixture of 10^6^ dead and 10^4^ viable *E. coli* O157:H7, the PMA real-time PCR assay accurately estimated viable cell amounts (Fig. 2). However, the assay over-estimated the numbers of *E. coli* O157:H7 when 10^3^ viable cells were measured in the presence of 10^6^ dead cells of the same strain (estimated log 3.5 CFU; Fig. 2).

### Quantification of Viable Cells of *E. coli* O157:H7 on Lettuce in the Growth Chamber

**Figure 3:**
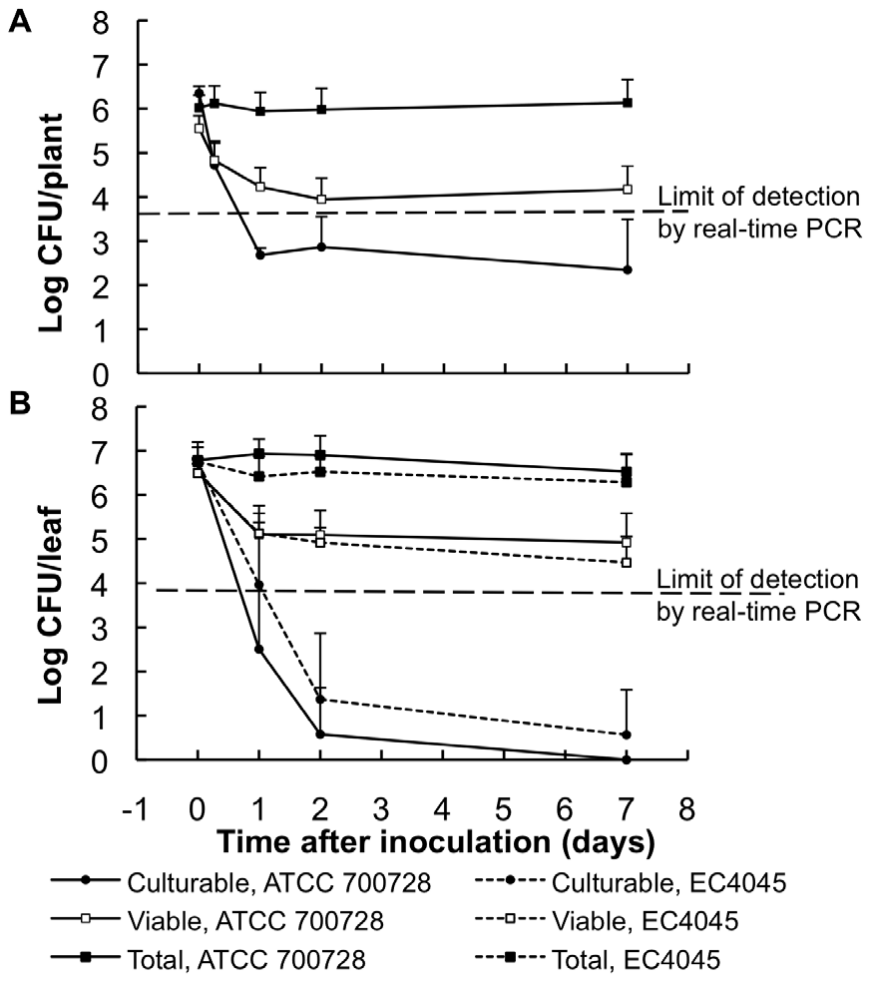
Survival of *E. coli* O157:H7 ATCC 700728 and EC4045 on inoculated lettuce in a growth chamber. *E. coli* O157:H7 cell amounts were measured by plate count (culturable), real-time PCR (total), and PMA real-time PCR (viable) methods. Plants were inoculated with a spray bottle (A) or by drop (B) and incubated at a relative humidity of 30%. The mean ± stdev of 15 lettuce plants or leaves at each sampling time for three replicate experiments is shown.

*E. coli* O157:H7 ATCC 700728 was applied to lettuce plants in the growth chamber either by spray or application of 2-µl drops directly onto the leaves. The latter was used specifically to prevent the hazards associated with aerosolization of the virulent strain EC4045 that can occur during spray application. The inoculum level was adjusted to deliver approximately 10^6^ CFU per plant by spray or per leaf by drop, and after inoculation, the plants were immediately incubated at low (30%) relative humidity and moderate daytime/nighttime temperatures of 22 and 18°C. With both inoculation methods, culturable *E. coli* O157:H7 ATCC 700728 cell populations declined to an average of 2.6±0.2 log CFU per leaf or plant within the first 24 h after inoculation (Fig. 3). For plants inoculated by the drop method, the number of culturable cells declined by 4 to 5 log CFU/leaf by day 2 to an average of 10 CFU/leaf (Fig. 3B). After inoculation by spray, the average number of culturable *E. coli* O157:H7 ATCC 700728 cells remained higher and ranged from 100 to 1000 CFU/plant, even on day 7 of the study (Fig. 3A). The cell numbers of strain EC4045 also declined rapidly after inoculation onto lettuce (Fig. 3B). Although there were significantly higher numbers of culturable EC4045 cells than strain ATCC 700728 cells at 24 h after inoculation, the culturable amounts of both strains declined to equivalently low levels within 48 h on lettuce (Fig. 3B).

Real-time PCR was used to quantify total *E. coli* O157:H7 cells on the lettuce. The number of *E. coli* O157:H7 cells remained equivalent to the inoculum cell density for 7 days, regardless of the strain or the inoculation method used (Fig. 3). The addition of PMA prior to real-time PCR amplification revealed that viable cell quantities were equal to the numbers of total and culturable cells at the time of inoculation but then declined by an average of 100-fold within the first day. For both ATCC 700278 and EC4045 strains, the number of viable cells detected using PMA real-time PCR remained above the detection limit and was significantly higher than estimated by the standard plate count method (Fig. 3).

### Quantification of Viable and Total *E. coli* on Lettuce in the Field

**Figure 4:**
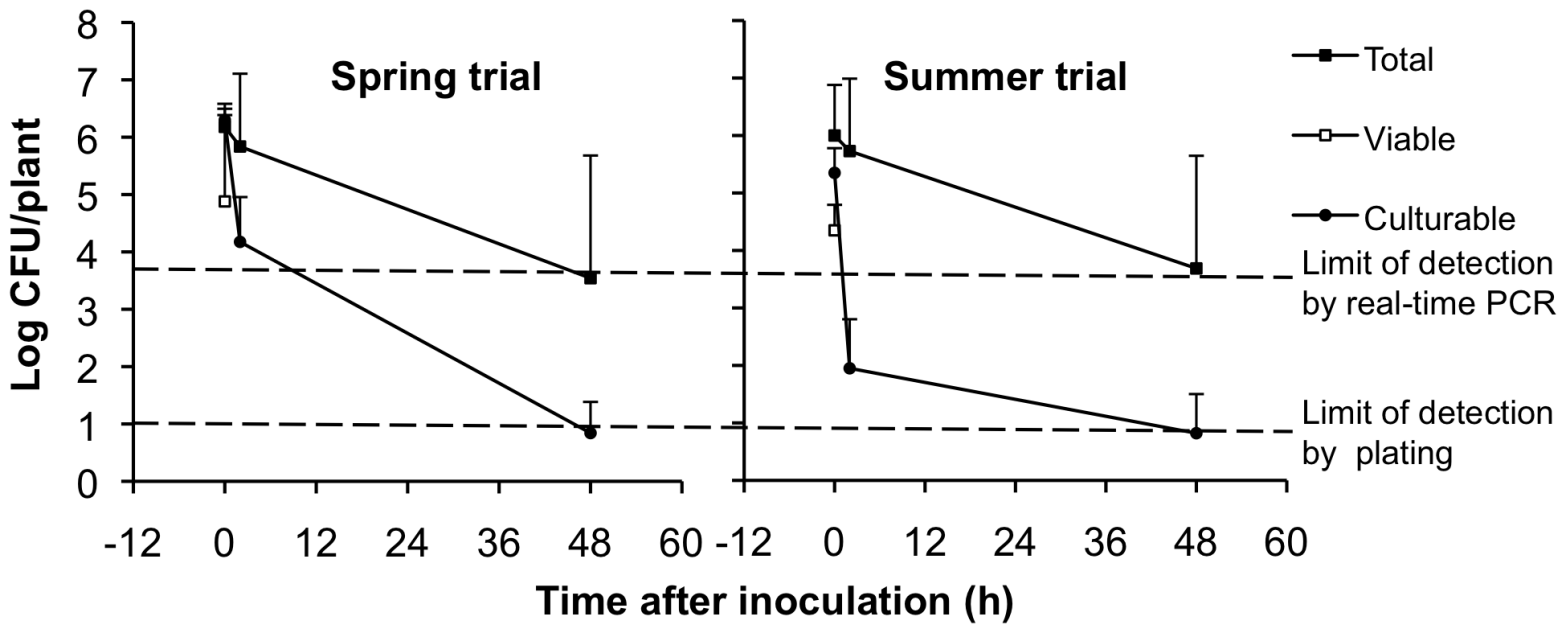
*E. coli* O157:H7 ATCC 700728 survival on lettuce in the field. *E. coli* O157:H7 amounts were measured by plate count (culturable), real-time PCR (total), and PMA real-time PCR (viable). Viable amounts were below the detection limit (3.7 log CFU/plant or leaf) at 2 and 48 h. The mean ± stdev of 12 lettuce plants at each sampling time is shown.

Lettuce plants in the field were spray inoculated with *E. coli* O157:H7 ATCC 700278 at a density of 10^6^ CFU/plant. *E. coli* O157:H7 was not detected on uninoculated (control) lettuce plants either by culture or by real-time PCR with or without PMA. Within 2 h after application onto the lettuce, culturable cell amounts declined by 2 and 4 log for plants inoculated in the spring and summer trials, respectively (Fig. 4). Two days after inoculation, the amounts of culturable *E. coli* ATCC 700278 were reduced to the lower limit of detection by plating for most plants (less than 10 CFU per plant). At that time, *E. coli* O157:H7 was not retrieved by plating or enrichment for 3% and 13% of the spring and summer plants, respectively.

In contrast to colony enumerations of *E. coli* O157:H7, total (viable and dead) cell numbers determined by real-time PCR quantification were equivalent to the inoculum levels 2 h after application to lettuce plants. Two days after application the numbers of *E. coli* O157:H7 estimated by real-time PCR decreased, on average, by 2 log CFU/plant and were below the limit of detection (3.7 log CFU/plant) for six out of 24 plants tested for both field trials.

PMA real-time PCR (viable cells) underestimated *E. coli* O157:H7 numbers by 1 log for plants collected at the time of *E. coli* O157:H7 ATCC 700278 inoculation. The average numbers of viable *E. coli* O157:H7 cells determined by this method were below the limit of detection (3.7 log CFU/plant) 2 h after inoculation. Hence, in contrast to the growth chamber studies, the utility of PMA real-time PCR was limited on field-grown plants. The finding was supported upon examination of lettuce heads collected from the field and spiked with known quantities of viable *E. coli* O157:H7 cells in the laboratory. PMA-qPCR underestimated the number of viable *E. coli* O157:H7 cells compared with CFU enumeration by approximately five-fold (data not shown).

## Discussion

Accurate measurements of pathogen amounts and viability are important for risk assessments and control mechanisms aimed at preventing outbreaks of foodborne illness. This study was the first to apply a culture-independent method to quantify the number of *E. coli* O157:H7 cells on plants inoculated with the (attenuated) pathogen in the field. We also compared the utility of the method for quantifying virulent and avirulent pathogen survival on laboratory-grown lettuce. The first step in the development of culture-independent methods for bacterial enumeration is to ensure that the technique is optimized for the specific organism and environmental conditions of interest [31]. While numerous assessment methods are available [25], [27], we selected PMA combined with real-time PCR because this technique offered the possibility of rapid enumeration of viable cells in a variety of environments. For *E. coli* O157:H7 ATCC 700728, we first identified appropriate real-time PCR primers, PMA concentrations, and PMA incubation times with a goal of selective and sensitive detection of viable *E. coli* O157:H7 cells in the presence of high levels of dead *E. coli* O157:H7 cells. Among the results was the confirmation that PMA-mediated PCR-inhibition is incomplete when the product size is less than 200 bp [28]–[30], [32]. Although our tests on PMA real-time PCR revealed some potential short-comings with the method, most notably the interference of high quantities of inactivated cells and the relatively high numbers of cells required for detection, these parameters were within the range found in other PCR-based studies [28]–[30], [33]–[35].

*E. coli* O157:H7 strains ATCC 700728 and EC4045 were inoculated onto leaves of potted lettuce plants and incubated in a growth chamber under temperature and low humidity conditions that favored the decline of cell numbers in trends similar to those recorded in field trials performed in the Salinas Valley. This approach differs from most other laboratory studies that examined plants exposed to high moisture and temperatures supportive of pathogen growth [4], [11], [36], [37]. Within 2 days after inoculation on lettuce plants, the number of *E. coli* O157:H7 ATCC 700728 cells able to form a colony on TSA declined 1000-fold when the pathogen was initially applied at a level of 6 log CFU per plant or per leaf using an aerosolized spray and declined >10^5^-fold when either strain was applied directly in 2-µl drops. The numbers of culturable cells remained similar for the subsequent 5 days, suggesting that the initial events after contact with the plant are crucial for determining the survival of the organism on lettuce.

In contrast with plate counts, real-time PCR targeting of the *E. coli* O157:H7 ATCC 700278 and EC4045 inoculants showed that the genomic DNA of the organisms remained on the lettuce in quantities nearly equivalent to the inoculum concentrations over the duration of the 7-day experiment. This finding confirmed that both strains adhered to the leaf surfaces and the recovery method was sufficient to remove the majority of the cell inoculants from the plant. Moreover, the results indicated the presence of high numbers of intact *E coli* O157:H7 cells on lettuce even when very few were able to form colonies on TSA.

Exposure of *E. coli* O157:H7 suspensions to PMA prior to real-time PCR quantification facilitated the detection of cells with an intact cell membrane, and these cells were likely viable. Application of this method to *E. coli* ATCC 700728 and EC4045 recovered from growth chamber lettuce indicated that a significant fraction of the pathogen inoculants were viable, even though colony-based assessments indicated otherwise. This finding was particularly evident for *E. coli* O157:H7 inoculated onto leaves by direct drop inoculation. While an average of 5 log CFU per leaf were viable as measured by PMA real-time PCR, less than 1 log CFU per leaf were enumerated by culturing. These results are supported by previous studies reporting that *E. coli* O157:H7 enters into a viable but not culturable (VBNC) state on lettuce leaves incubated at cold (4 to 16°C) temperatures [27]. Similar outcomes were reported for other *E. coli* including *E. coli* O104:H4 in water or after exposure to toxic concentrations of copper [38], [39]. Once the stress condition(s) was removed, a fraction of the cells recovered the capacity to grow in laboratory media, thereby indicating a potential of the *E. coli* O1O4:H4 cells to cause human disease [38]. The formation of VBNC bacterial cells on plants also was previously described for *Listeria monocytogenes* on parsley [25]. The number of viable *L. monocytogenes* cells was 1 to 2 log higher than the culturable cells recovered from parsley grown in greenhouses at 20°C under low relative humidity (between 47 to 69% RH); growth of the VBNC cells was not restored on the plants when the RH was increased to 100% [25]. Although we did not examine for *E. coli* O157:H7 recovery from the VBNC-like state, future efforts might investigate whether those cells can recover and resume growth either under growth-conducive conditions on lettuce or after removal of the cells from the plants and prior to or after plating for viable cell enumeration.

Overall, the toxigenic strain EC4045 survived in similar quantities as ATCC 700728 on lettuce. A recent study showed that certain lineages of *E. coli* are more commonly associated with plants and presumably have evolved the capacity to tolerate plant-associated environments better than *E. coli* isolated from other sites [40]. Because we only compared two strains, subsequent investigations should examine multiple attenuated and virulent O157:H7 strains isolated from different sites (e.g. plants and cattle) for their capacity to colonize and persist on lettuce under field-relevant conditions.

Survival of *E. coli* O157:H7 on lettuce also was measured in two field studies. Culturable amounts of strain ATCC 700728 declined shortly after inoculation onto plants in the field, as we reported previously [14]. Rates of cell decline were similar to *E coli* O157:H7 on lettuce in the growth chamber directly inoculated in drops with a pipette. Real-time PCR estimates of *E. coli* O157:H7 ATCC 700728 in lettuce leaf washes showed that this strain was present on the plants immediately after inoculation and 2 h later in quantities equivalent to the inoculum levels. Importantly, the rapid decline in culturable *E. coli* during the first hours after application onto plants in the field was not due to an inability to remove the organism from the lettuce or from dispersal and lack of strain attachment. Rather, it appears that the majority of the *E. coli* cells in the inoculum either died shortly after application or entered a VBNC state.

In contrast to the growth chamber experiment results, the numbers of *E. coli* O157:H7 cells were below detection by real-time PCR within 2 days after inoculation onto field lettuce. These findings suggest that the *E. coli* O157:H7 cells and genomic DNA were degraded rapidly. Environmental parameters such as solar radiation, heat, and water stress could be responsible for the differences in the stability of *E. coli* O157:H7 DNA in the field compared with the laboratory. Alternatively, cell maintenance might depend on other microorganisms on the leaves [41]. Also, it is notable that different lettuce cultivars were used in the field and growth chamber studies, which may have impacted survival. The potential for cultivar-dependent effects was shown for *E. coli* O157:H7 on lettuce cultivars grown under axenic conditions in the laboratory [42].

Because the *E. coli* O157:H7 ATCC 700728 DNA was degraded on field-grown plants within 2 days after inoculation, it is unlikely that the organism developed a VBNC state, particularly over longer time scales (days and weeks). However, this possibility could not be directly addressed using the PMA real-time PCR assay in the field trials. Viable cell amounts measured by culturing and PMA real-time PCR were in agreement immediately after application of ATCC 700728 onto laboratory-grown lettuce, but PMA-mediated detection was impaired on plants from the field. For those plants, the viable cell number estimates for strain ATCC 700278 were 10-fold lower as measured by PMA real-time PCR than by colony enumeration and total cell numbers estimated by real-time PCR. These differences might have been due to the higher turbidity or opacity of the lettuce plant washes from the field samples, thereby preventing light from penetrating the suspension during the PMA photoinactivation step. This interference would prevent the inactivation of free (unbound) PMA, resulting in sufficient quantities of the compound to bind genomic DNA released from viable cells during the subsequent DNA extraction and amplification steps. In addition, the PMA real-time PCR assay was unable to detect low numbers of cells. Attempts to detect the ATCC 700278 strain after concentrating the leaf washes were unsuccessful (data not shown). Similarly, PMA real-time PCR was found to be more reliable for viable cell detection in diluted wastewater than in pooled and concentrated wastewater samples [32]. Such factors strongly limit the overall usefulness of this approach for field-grown plants. However, this method is informative for examination of *E. coli* O157:H7 on “cleaner” plants grown in the growth chamber and not exposed to the (a)biotic conditions that are common outdoors.

In conclusion, this study illustrates the similarities and differences between controlled studies of human pathogens on plants in a growth chamber and experiments examining the population dynamics of pathogens on plants under production-like conditions in the field. By applying relevant environmental conditions (moderate temperature and low RH) and droplet inoculation in the growth chamber, we were able to more closely mimic the rapid decline in *E. coli* O157:H7 culturability that was observed after inoculation of this organism onto lettuce plants in the Salinas Valley [14]. Culture-independent assessments confirmed that the pathogen remains on the plant long after application. However, field studies showed that at least for the majority of *E. coli* O157:H7 cell inoculants, the loss in culturability was most likely due to cell death rather than an inability to form colonies on standard laboratory media. Hence, this work confirmed our observations that low numbers of *E. coli* O157:H7 persist on lettuce grown in the Salinas Valley and variations in pathogen survival among individual plants are dependent on other unknown factors (e.g. location on the leaf, composition of the indigenous microbiota, and plant position in the field).

## Supporting Information

Figure S1 **Effect of increasing PMA concentration on detection of live and dead cells by real-time PCR.** Live or isopropanol-inactivated dead cells of *E. coli* O157:H7 ATCC 700728 (5×10^6^ CFU) were incubated for 30 min in 0, 50, or 100 µM PMA prior to real-time PCR with the lpfA primer set. Each bar represents the mean ± stdev for three independent replicates.
(DOCX)

Figure S2 **Standard curves for detection of** ***E. coli*** **O157:H7 with hlyA primers (A) and lpfA primers (B)**. DNA was extracted by boiling as described in the Materials and Methods from a stationary phase culture of *E. coli* O157:H7 diluted to a cell density of 10^6^ CFU/ml. Real-time PCR was performed for each dilution in triplicate. The limit of detection for *E. coli* O157:H7 cells with the lpfA and hlyA primers was 100 CFU/ml and 1000 CFU/ml, respectively. The amplification efficiency was 95% for EC4045 and 100% for ATCC700728 with the lpfA primers and 84% for ATCC700728 with the hlyA primers.
(DOCX)

Table S1 **Primers used in this study.**
(DOCX)

Table S2 **Ct values for real-time PCR measurements performed using different annealing temperatures and gene targets of** ***E. coli*** **O157:H7 ATCC700728 and** ***E. coli*** **K12.**
(DOCX)
