# Supplementary figures and images for: Assessments of Total and Viable Escherichia coli O157:H7 on Field and Laboratory Grown Lettuce
Source: PLoS One. 2013 Jul 30;8(7):e70643. doi: 10.1371/journal.pone.0070643 (PMC3728298; doi:10.1371/journal.pone.0070643)

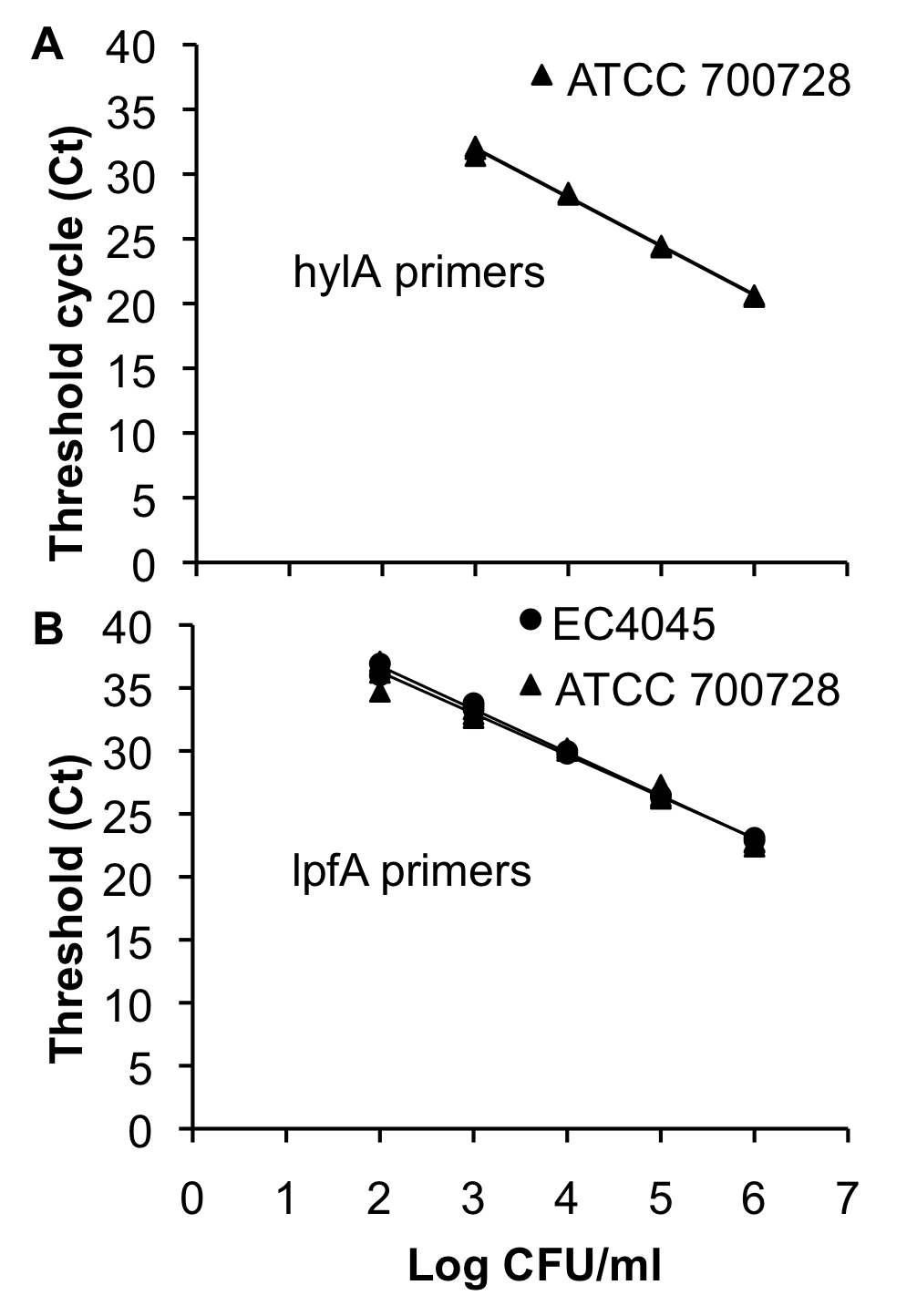

Supplement: Figure S2 — Standard curves for detection of E. coli O157:H7 with hlyA primers (A) and lpfA primers (B). DNA was extracted by boiling as described in the Materials and Methods from a stationary phase culture of E. coli O157:H7 diluted to a cell density of 106 CFU/ml. Real-time PCR was performed for each dilution in triplicate. The limit of detection for E. coli O157:H7 cells with the lpfA and hlyA primers was 100 CFU/ml and 1000 CFU/ml, respectively. The amplification efficiency was 95% for EC4045 and 100% for ATCC700728 with the lpfA primers and 84% for ATCC700728 with the hlyA primers. (DOCX) [file pone.0070643.s002.docx]
