# Supplementary material for: Assessments of Total and Viable Escherichia coli O157:H7 on Field and Laboratory Grown Lettuce
Source: PLoS One. 2013 Jul 30;8(7):e70643. doi: 10.1371/journal.pone.0070643 (PMC3728298; doi:10.1371/journal.pone.0070643)
Supplement: Table S1 — Primers used in this study. (DOCX) [file pone.0070643.s003.docx]

**Table S1. Primers used in this study.**

| **Target gene** | **Sequence (5’ to 3’)** |
| --- | --- |
| *csgA* ^a^ | csgA-F: GATGTTGGTCAGGGCTCAG |
|  | csgA-R: CCACCGAATTGTTTAACTGTC |
| *eae* ^a^ | eae-F: TCTGTGTGGATGGTAATAAATTTTTG |
|  | eae-R: GTAAGTTACACTATAAAAGCACCGTCG |
| *espA* ^a^ | espA-F: GATGTTGGTCAGGGCTCAG |
|  | espA-R: ATCTAAAGCGTCAACCACGG |
| *fliC* ^a^ | fliC-F: TCGTCAAGTTGCCTGCATC |
|  | fliC-R: TTAGCTGCCACCCTTCATG |
| *ler* ^a^ | ler-F: GTAAACACCTTTCGATGAGTTCC |
|  | ler-R: GAGTCGATTCAGAAGCAGATTAC |
| *lpfA* ^a^ | lpfA-F: CACCGTTAAGAGCGACCAGGG |
|  | lpfA-R: GAAGATTGCGATACCACCACG |
| *hlyA* ^b^ | hlyA-a: GAGTCGATTCAGAAGCAGATTAC |
|  | hlyA-b: TACGGGTTATGCCTGCAAGTTCAC |

^a^ Primer sequences in the enterocyte effacement loci were obtained from Sharma *et al.* [20] and ^b^ in the plasmid-encode enterohemolysin from Wang *et al.*[21].
