# Supplementary material for: Assessments of Total and Viable Escherichia coli O157:H7 on Field and Laboratory Grown Lettuce
Source: PLoS One. 2013 Jul 30;8(7):e70643. doi: 10.1371/journal.pone.0070643 (PMC3728298; doi:10.1371/journal.pone.0070643)
Supplement: Table S2 — Ct values for real-time PCR measurements performed using different annealing temperatures and gene targets of E. coli O157:H7 ATCC700728 and E. coli K12. (DOCX) [file pone.0070643.s004.docx]

**Table S2. Ct values for real-time PCR measurements performed using different annealing temperatures and gene targets of *E. coli* O157:H7 ATCC700728 and *E. coli* K12**.

| **Gene target** | **Product size** | **Strain** | **Ct Value** ^a^ | | | | | |
| --- | --- | --- | --- | --- | --- | --- | --- | --- |
|  |  |  | **Annealing temperature (°C)** | | | | | |
|  |  |  | **60** | **62** | **63** | **65** | **66** | **68** |
| *csg* | 121 | 700728 | 18.2 | 18.4 | 18.5 | 21.9 | 24.2 | n/a ^b^ |
|  |  | K12 | 24.0 | 26.6 | 30.4 | n/a^c^ | n/a | n/a |
| *eae* | 105 | 700728 | 17.7 | 17.9 | 18.5 | 21.7 | 24.1 | n/a |
|  |  | K12 | 36.8 | 36.5 | 36.9 | n/a | n/a | n/a |
| *espA* | 110 | 700728 | 16.7 | 17.4 | 17.2 | 18.9 | 19.9 | 26.9 |
|  |  | K12 | 34.1 | 34.1 | 33.9 | 35.9 | 37.1 | n/a |
| *fliC* | 132 | 700728 | 17.5 | 17.3 | 17.1 | 17.3 | 17.6 | 18.8 |
|  |  | K12 | 34.6 | 33.8 | 33.9 | 34.2 | 35.2 | 34.9 |
| *lerR* | 110 | 700728 | 17.8 | 17.9 | 18.2 | 19.4 | 20.6 | 27.4 |
|  |  | K12 | 35.6 | 37.0 | 36.35 | 39.68 | n/a | n/a |
| *lpfA* | 165 | 700728 | 16.5 | 16.8 | 16.9 | 17.8 | 18.6 | 21.3 |
|  |  | K12 | 35.1 | 35.8 | 35.2 | 35.8 | 37.7 | n/a |

^a^ Real-time PCR amplification was performed using 1 ng of pure genomic DNA

^b^ n/a = no amplification
