# Supplementary material for: Assessments of Total and Viable Escherichia coli O157:H7 on Field and Laboratory Grown Lettuce
Source: PLoS One. 2013 Jul 30;8(7):e70643. doi: 10.1371/journal.pone.0070643 (PMC3728298; doi:10.1371/journal.pone.0070643)
Supplement: Supplementary file 4 [file pone.0070643.s001.docx]

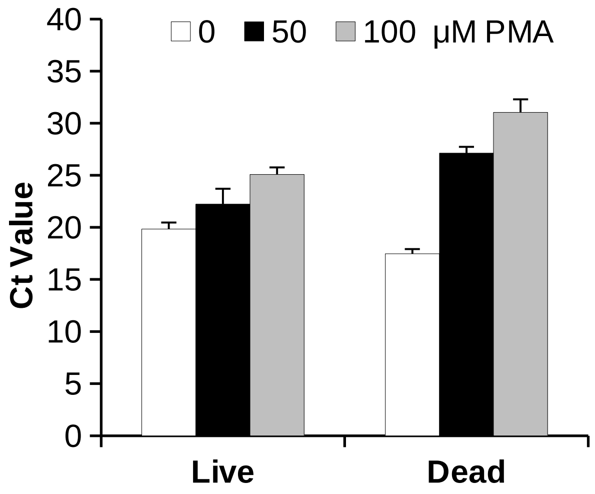

0
50
100 µM PMA
Ct Value
40
35
30
25
20
15
10
5
0
Live
Dead
